# Influence of intrastromal corneal ring implantation on intraocular pressure measurements using different tonometers in keratoconic eyes

**DOI:** 10.5935/0004-2749.2024-0217

**Published:** 2025-06-24

**Authors:** Christiano Scholte, Júlia Maggi Vieira, Leonardo Torquetti, Fábio Nishimura Kanadani

**Affiliations:** 1 Núcleo de Excelência em Oftalmologia, Belo Horizonte, MG, Brazil; 2 Glaucoma Institute, Belo Horizonte, MG, Brazil; 3 Centro de Excelência em Oftalmologia, Pará de Minas, MG, Brazil

**Keywords:** Keratoconus, Intraocular pressure, Cornea, Corneal stroma, Postoperative period, Tonometry ocular, Prostheses and implants

## Abstract

**Purpose:**

This study aimed to evaluate the influence of intrastromal corneal ring segment implants on the intraocular pressure measurements using Goldmann applanation tonometry, rebound tonometry, and noncontact tonometry in keratoconic corneas and analyze the intertonometer agreement.

**Methods:**

We enrolled 74 eyes in this observational and prospective study. Each participant had a complete eye examination, corneal analysis with Scheimpflug Tomography (Pentacam^®^), and intraocular pressure evaluation with Goldmann applanation tonometry, rebound tonometry, and noncontact tonometry, before and after intrastromal corneal ring segment implantation (on postoperative days 1, 7, 45, and 90). Intertonometer agreement was assessed using Bland-Altman analysis.

**Results:**

The mean age was 29.9 ± 10.2 years, and 47 (63.5%) eyes had keratoconus grade II. Intraocular pressures were higher for noncontact tonometry preoperatively and on 90 postoperative day (mean ± SD: 12.4 ± 2.2 and 12.1 ± 2.2 mmHg, respectively), followed by Goldmann applanation tonometry (11.1 ± 3.0 and 11.2 ± 2.7 mmHg, respectively), and were lower for rebound tonometry (9.7 ± 3.4 and 9.4 ± 3.2 mmHg, respectively). The variation from the Goldmann tonometry on 7 postoperative day to the baseline (p=0.022) and that of noncontact tonometry on 90 postoperative day to the baseline (p=0.021) were statistically significant. The rebound tonometry underestimated intraocular pressure when compared with the Goldmann applanation tonometry by a mean of 1.47 ± 5.19 mmHg. Noncontact tonometry, when compared with Goldmann applanation tonometry, overestimated intraocular pressure by a mean of 1.23 ± 4.15 mmHg.

**Conclusion:**

Despite statistically significant differences between some postoperative periods, the intraocular pressure measurement differences may not be clinically relevant.

## INTRODUCTION

Glaucoma is the leading cause of irreversible blindness worldwide^(1)^. Although not considered the cause of glaucoma, intraocular pressure (IOP) alone is the main and only modifiable risk factor. An accurate and reliable IOP measurement is essential for monitoring the disease and evaluating treatment effectiveness^(2)^. Randomized clinical trials showed that it is related to lower progression or worsening of glaucoma rates over 5 years^(3^,^4)^.

For more than 50 years, the Goldmann applanation tonometer (GAT) has been the gold standard device for IOP measurement^(5)^. Goldmann and Schmidt’s calculations for GAT were based on Imbert-Fick’s law, assuming a spherical, dry, perfectly flexible, and infinitely thin surface as an ideal cornea^(6)^. Thus, the GAT principle has been reported to be influenced by the cornea’s biophysical properties, such as central thickness (CCT), curvature (CC), astigmatism (CA), and hysteresis (CH) despite performing the measurements with accuracy^(7^-^9)^. Alternative methods, such as new noncontact and mixed tonometric systems (Rebound), have been proposed to overcome these limitations^(10)^.

Significant changes in IOP measured by GAT have been demonstrated not only in diseased corneas (dystrophies and ectasias) but also after keratorefractive procedures and corneal transplantation, lamellar or full thickness, making its use also limited^(11)^. Despite this exhaustive demonstration, only a few studies have evaluated the influence of intrastromal corneal ring segment (ICRS) implantation on IOP reading by using different tonometric systems.

The ICRS is one of the surgical options for keratoconus treatment. It has promoted visual rehabilitation through corneal remodeling and tension redistribution^(12)^, improving vision by reducing refractive errors, mean corneal curvature, and asphericity^(13)^. In addition, the ICRS implant is a surgical alternative to delay, if not eliminate, the need for lamellar or penetrating keratoplasty in primary or secondary corneal ectasias^(14)^. Unlike subtraction techniques, such as corneal refractive surgery with the excimer laser, ICRS modifies the cornea without tissue removal. Since 2000, studies have reported successful results with these implants in keratoconic eyes, following different nomograms^(15)^.

The study aimed to evaluate the influence of the ICRS implantation on IOP measurement and compare these measurements among three different tonometric systems: Goldmann’s applanation, rebound (RbT), and noncontact tonometer (NCT).

## METHODS

We conducted an observational, prospective study at the *Núcleo de Excelência em Oftalmologia* in Belo Horizonte, MG, Brazil, between April 2019 and April 2021. The Ethics Committee of Santa Casa BH, Minas Gerais, Brazil, approved the study protocol, and all patients provided informed consent before the study.

All participants underwent a complete ophthalmic examination, including best-corrected visual acuity, slit--lamp biomicroscopic, and fundoscopic examination. Wearing of contact lens was suspended at least 1 week before the examination. An experienced corneal specialist (CS) diagnosed keratoconus, defined by clinical examination (slit-lamp biomicroscopy signs) and confirmed tomographically by using a rotating Scheimpflug camera (Oculus Pentacam, Wetzlar, Germany) before all IOP measurements.

The individuals were included if they have keratoconus grades I to IV, low visual acuity without improvement with refraction, intolerance to rigid gas permeable contact lenses, and age >10 years old. Exclusion criteria were presence of corneal opacity at the corneal apex, stromal scars, hydrops, corneal thickness <300 micra in the ICRS track, severe ocular atopy, active local or systemic infection, presence of previous ocular disease (glaucoma or retinal diseases) that compromise visual acuity, current use of eye hypotensive therapy or topical steroids, history of corneal disease or other intraocular pathology, previous eye surgery, uveitis, and nystagmus.

In this study, 25-images-per-scan mode and auto-mea-surement mode were selected with Pentacam^®^. The same experienced examiner (CS) performed all measurements in both eyes and under dim lighting conditions.

After Pentacam^®^ evaluation, the same examiner (CS) measured the IOP as follows: RbT, NCT, and GAT, always in the same order, between 2:00 and 4:00 pm. GAT was used for the last IOP measurement to avoid corneal--compression-induced aqueous outflow increase that would affect subsequent IOP readings. Each tonometer was calibrated based on the manufacturer’s guidelines before its use. A 3-min interval between measurements and the same tonometers were used throughout the study.

### Tonometry sequence

For the RbT (Icare iC100; Tiolat Oy), one practitioner performed all measurements based on the manual.

After RbT measurements, tonometry was measured using NCT (Topcon CT-80; Topcon Corporation, Tokyo, Japan) that automatically recorded three IOP readings, with their average per eye recorded.

Thereafter, applanation tonometry was performed using a Goldmann tonometer (Haag-Streit, Koeniz, Switzerland). Two measurements were performed for each eye, and the mean value was considered for statistical analysis. To reduce any bias, the tonometer dial was reset before each measurement and covered. Another observer who recorded the results performed the IOP reading.

### Surgical technique

The FerraraRing^©^ (AJL Ophthalmic, Vitória, Spain) 5.0 mm ICRS was implanted in all patients centered on the first Purkinje reflex. The same surgeon (CS) performed all surgical procedures using a standard mechanical technique that has been previously described^(16)^. The selection of the number (1 or 2), arc length, and ICRS thickness was based on the nomogram defined by the manufacturer (4th and 5th generation). After the procedure, all patients were prescribed topical dexamethasone 0.1% (Maxidex^®^, Alcon) for 21 days (tapering), gatifloxacin 0.3% (Zymar^®^, Allergan) 4 times daily for 7 days, and hypromellose drops (Fresh Tears®, Allergan) every 6 h for 30 days. The patients were followed up on postoperative day (POD) 1, 7, 45, and 90.

### Statistical analysis

IOP results were compared between GAT, RbT, and NCT.

Data were analyzed using the statistical package SPSS version 20.0 (SPSS Inc, Chicago, IL) and Excel 2013 (Microsoft, Redmond, WA).

Descriptive statistics included measures of central tendency (mean and median) and dispersion (standard deviation) for continuous variables. For categorical variables, the frequency and percentage for each category were calculated. Continuous variables with normal and non-normal distributions were compared using independent samples paired t-test and Wilcoxon test, respectively. Categorical data were analyzed using the chi-square test. Altman and Bland plot was used to assess agreement. Significance level was established at p<0.05 and 95% confidence interval (CI).

## RESULTS

We included 74 eyes from 74 keratoconic individuals. The mean age was 29.9 ± 10.2 years, of which 47 (63.5%) were keratoconus grade II, and 70.3% received only one ICRS. Table 1 shows the demographic details and descriptive statistics (Table 1).

**Table 1:** Demographic data and performed implant characteristics

Variables	n=74	%
Operated eye		
Right	36	48.6
Left	38	51.4
Sex		
Male	25	33.8
Female	49	66.2
Age (years)		
Average (DP)	29.9 ( ± 10.2)	
Median (Min-Max)	29 (10;58)	
Keratoconus staging		
I	13	17.6
II	47	63.5
III	11	14.9
IV	3	4.0
Preoperative Visual acuity (logMAR)		
Average (SD)	0.4 ( ± 0.2)	
Median (Min-Max)	0.4 (0.2;1.3)	
Postoperative Visual acuity (logMAR)		
Average (SD)	0.2 ( ± 0.1)	
Median (Min-Max)	0.2 (0.0;0.5)	
Number of implanted segments		
One	52	70.3
Two	22	29.7
Implanted volume (mm^3^)		
Average (SD)	0.748 ( ± 0.246)	
Median (Min-Max)	0.143 (0.468;1.374)	

SD= standard deviation, Min= minimum, Max= maximum, logMAR= logarithm of the minimum angle of resolution, mm^3^: cubic millimeters.

The tonometric measurements of the GAT, RbT, and NCT were performed preoperatively and at POD 1, 7, 45, and 90 for operated and nonoperated eyes (Table 2). Overall, IOPs were higher in NCT preoperatively and on POD 90 (mean ± SD: 12.4 ± 2.2 and 12.1 ± 2.2 mmHg, respectively), followed by GAT (11.1 ± 3.0 and 11.2 ± 2.7 mmHg, respectively), and lower in RbT (9.7 ± 3.4 and 9.4 ± 3.2 mmHg, respectively).

**Table 2:** Tonometric measurements in operated and nonoperated eyes

Variables	n=74 eyes			
	Operated eye		Nonoperated eye	
	Mean (SD)	Median (MIn-Max)	Mean (DP)	Median (MIn-Max)
Goldmann tonometry				
Preoperative (mmHg)	11.1 ( ± 3.0)	11 (6;22)	11.7 ( ± 2.7)	12 (7;22)
POD 1 (mmHg)	11.4 ( ± 3.2)	11.5 (5;20)	11.5 ( ± 2.9)	11 (6;21)
POD 7 (mmHg)	12.0 ( ± 3.4)	12 (6;22)	11.9 ( ± 3.1)	12 (6;21)
POD 45 (mmHg)	11.2 ( ± 2.8)	12 (6;19)	11.4 ( ± 2.7)	11 (6;19)
POD 90 (mmHg)	11.2 ( ± 2.7)	11 (6;19)	11.5 ( ± 2.6)	12 (6;19)
Rebound tonometry				
Preoperative (mmHg)	9.7 ( ± 3.4)	9 (5;19)	10.2 ( ± 3.3)	9 (5;17)
POD 1 (mmHg)	9.7 ( ± 3.7)	8 (4;18)	10.2 ( ± 3.5)	10 (4;20)
POD 7 (mmHg)	10.3 ( ± 3.7)	10 (4;21)	10.6 ( ± 3.6)	10 (5;18)
POD 45 (mmHg)	9.4 ( ± 3.4)	8 (5;19)	10.4 ( ± 3.5)	9,5 (5;19)
POD 90 (mmHg)	9.4 ( ± 3.2)	9 (4;18)	10.4 ( ± 3.4)	9 (4;18)
Noncontact tonometry				
Preoperative (mmHg)	12.4 ( ± 2.2)	12 (8;19)	12.9 ( ± 2.4)	13 (8;19)
POD 1 (mmHg)	12.5 ( ± 2.6)	12 (6;20)	13.2 ( ± 2.3)	13 (8;18)
POD 7 (mmHg)	12.7 ( ± 2.7)	12 (7;21)	13.0 ( ± 2.4)	13 (8;19)
POD 45 (mmHg)	12.0 ( ± 2.4)	12 (7;19)	12.9 ( ± 2.3)	13 (9;20)
POD 90 (mmHg)	12.1 ( ± 2.2)	12 (6;18)	13.2 ( ± 2.4)	13 (9;19)

POD= postoperative day; SD= standard deviation; Min= minimum; Max= maximum; Δ= variation; mmHg= millimeters of mercury.

Comparisons between variations in measurements (Δ) for different postoperative periods (POD 1, 7, 45, and 90) to the baseline were obtained by using different tonometers (GAT, RbT and NCT) between operated and nonoperated eyes.

Only two of the comparisons had a significant p-value. Table 3 shows the variation from the GAT to POD 7 to the baseline (p=0.022) and that of NCT to POD 90 to baseline (p= 0.021).

**Table 3:** Comparison between variations in measurements of different tonometries and postoperative periods between operated and nonoperated eyes

Variables	Operated eye	Nonoperated eye	Difference (CI95%)	p^a^
	Mean (SD)	Mean (SD)		
**Goldmann tonometry variation (mmHg)**				
Δ POD 1 to baseline	0.2 ( ± 2.1)	-0.2 ( ± 2.1)	0.4 (-0.1;1.0)	0.143
Δ POD 7 to baseline	0.9 ( ± 2.9)	0.1 ( ± 2.3)	0.7 (0.1;1.4)	0.022^*^
Δ POD 45 to baseline	0.0 ( ± 2.1)	-0.3 ( ± 1.6)	0.3 (-0.1;0.8)	0.172
Δ POD 90 to baseline	0.0 ( ± 1.9)	-0.3 ( ± 2.0)	0.3 (-0.2;0.9)	0.211
**Rebound tonometry variation (mmHg)**				
Δ POD 1 to baseline	0.0 ( ± 2.1)	-0.0 ( ± 2.1)	-0.0 (-0.6;0.5)	0.886
Δ POD 7 to baseline	0.6 ( ± 1.8)	0.4 ( ± 1.9)	0.2 (-0.3;0.7)	0.408
Δ POD 45 to baseline	-0.2 ( ± 1.9)	0.2 ( ± 2.0)	-0.4 (-1.0;0.1)	0.106
Δ POD 90 to baseline	-0.2 ( ± 1.7)	0.2 ( ± 1.7)	-0.4 (-0.9;0.1)	0.137
**Noncontact tonometry variation (mmHg)**				
Δ POD 1 to baseline	0.2 ( ± 1.6)	0.3 ( ± 1.9)	-0.1 (-0.6;0.4)	0.697
Δ POD 7 to baseline	0.4 ( ± 2.0)	0.0 ( ± 1.8)	0.3 (-0.1;0.8)	0.207
Δ POD 45 to baseline	-0.,3 ( ± 1.8)	0.0 ( ± 2.0)	-0.4 (-0.8;0.1)	0.114
Δ POD 90 to baseline	-0.2 ( ± 1.6)	0.3 ( ± 1.7)	-0.5 (-0.9;-0.1)	0.021^*^

POD= postoperative day; SD= standard deviation; 95% CI= 95% confidence interval; Δ= variation; mmHg: millimeters of mercury.

a Paired Student *T-Test.*

* **Significant p-values.**

Keratoconus stage, number or volume of implanted corneal segments, and IOP variations showed no significant correlation from baseline to POD 90 (Table 3).

**Figure 1:**
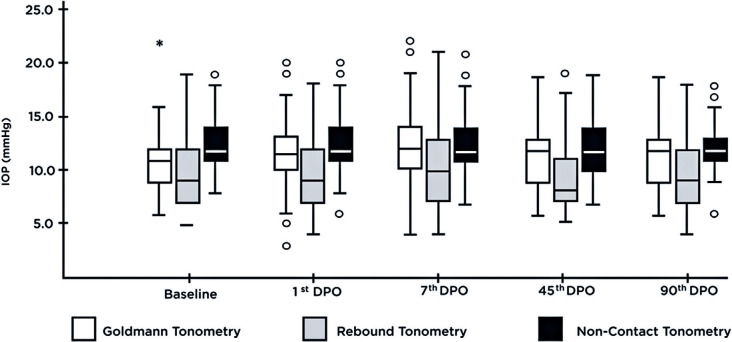
Boxplot of Goldmann, rebound, and noncontact tonometry (mmHg) measurements over time.

Boxplot graphs from raw of GAT, RbT, and NCT measurements demonstrated the behavior of data distribution. The NCT (although the median value was similar between the postoperative periods) and RbT boxplots showed high dispersion (represented by the interquartile range) and high amplitude of the data in addition to an asymmetry of its distribution and the presence of outliers at all times. Conversely, in the GAT boxplot, although the median locations increased and declined, no data dispersion was as intense as in the other tonometers, in addition to the data set being more symmetrical (Figure 1).

The Bland-Altman plot of IOP recorded by GAT and RbT shows a mean difference of +1.47 mmHg; the upper and lower limits of agreement were +6.66 and -3.71 mmHg, respectively. This shows a fixed systematic bias (GAT recorded a higher IOP consistently than RbT, but the difference did not change with increasing IOP).

**Figure 2:**
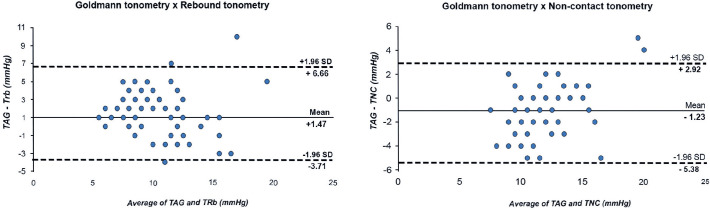
Bland-Altman plots for GAT and other tonometers (RbT and NCT). The mean values and 95% LOA are indicated by solid and dashed lines, respectively.

The mean difference between GAT and NCT was -1.23 mmHg, and the limits of agreement were +2.92 and -5.38 mmHg (Figure 2).

## DISCUSSION

The easy, safe, and accurate IOP measurement is essential for glaucoma diagnosis and follow-up. Obtaining an accurate IOP measurement has always been an important problem in patients with keratoconus because obtaining an accurate IOP measurement is difficult mainly due to corneal thickness, biomechanical properties, and corneal surface deformation^(17)^.

Although some cross-sectional articles have already evaluated the correlation between ICRS implantation and IOP measurement^(18^,^19)^, this study had a prospective design, with a more detailed analysis of preand postoperative IOP measurements.

GAT IOP was statistically significantly higher in eyes with ICRS on POD 7, and the IOP NCT was lower on POD 90 than in control eyes. Although these IOP measurements could be postulated as secondary to the effect of steroid use in the postoperative period, all tonometers should demonstrate the same tendency even at low concentrations and for a short period^(20)^.

Therefore, this IOP variation may be partially explained, particularly as boxplot graphs revealed how the NCT and RbT data showed greater variability (greater dispersion and high amplitude) and asymmetry of their distribution. Possibly, this may have caused the statistical significance of the NCT finding.

Despite these differences, this difference in GAT and NCT of +0.7 (p=0.022, 95% CI: 0.2-0.9 mmHg) and -0.5 (p=0.021, 95% CI: -0.9 to -0.1), respectively, is not clinically meaningful.

Contrary to expectations, no interference was observed between the independent variables (keratoconus stage, number, or volume of implanted corneal segments) and variations in tonometric measurements.

Our results coincide with the only prospective work by Arribas-Pardo wherein no clinical changes were found in IOP measurements using all tonometers (GAT, Icare Pro, Tonopen XL, dynamic contour tonometer, and ORA) after ICRS implantation^(21)^.

Conversely, studying IOP in Intacs eyes, Ruckhofer et al. found that the IOP measured using GAT was lower than baseline at all postoperative examinations, decreasing to a maximum of -1.75 ± 2.93 mmHg at 6 months^(22)^. However, this difference was not clinically meaningful despite the statistical difference.

Clinical decision-making requires the use of evidence-based medicine through the integration of clinical expertise with systematic research. This is particularly important when evaluating changes in IOP, where one of the difficulties in clinical practice is differentiating the test-retest variability (TRV) measure from a real change in IOP. TRV measures reproducibility at different time intervals by administering the same test to the same participants at different times. All methods used to measure IOP have some level of TRV that should be considered^(5)^. The measured IOP can also differ in the short term through circadian variations in the long term through natural physiological variations^(23)^ or from changes related to the instrument or operator.

The interpretation of some published guidelines for IOP variability by GAT might be difficult. Under ideal conditions, the 95% CIs for intraand interobserver variability are 2.5 and 4 mmHg, respectively, but these values may be higher in clinical practice^(24)^.

### Interdevice agreement among the tonometers

GAT remains the gold standard tonometer, and agreements between other tonometers and GAT are important in clinical practice. Our results showed that the mean difference and 95% limits of agreement (LOAs) between GAT-RbT and GAT-NCT were +1.47 and 10.34 mmHg and -1.23 and 8.31 mmHg, respectively, indicating that IcCare® constantly underestimated GAT over the range of measured IOP, whereas NCT overestimated GAT. The 95% LOA demonstrates a relatively large range of differences between methods, possibly precluding the use of interchangeable GAT, RbT, and NCT, but using the same tonometer consistently during clinical follow-up is almost as important as the tonometer choice.

Our data agree with findings by Demirci et al. who compared RbT and NCT with GAT in three different age groups of normal patients^(25)^. NCT measurements were significantly greater than GAT and RbT in all groups, but no statistical difference was found between RbT and GAT measurements in the three groups although GAT was slightly higher than RbT. In a study comparing IOP measurements obtained with RbT and GAT in keratoconic corneas, Özcura et al.^(17)^ found that RbT measurements were on average 2.38 mmHg lower than GAT measurements and found a significantly positive correlation between CCT and IOP measurements in GAT and RbT, with RbT being more sensitive to decreased CCT than GAT.

Arribas-Pardo et al. also evaluated the agreement between GAT and RbT in corneas after ICRS implantation, and mean IOP measurements were lower with Icare^®^ than with GAT, with the difference always <2 mmHg^(26)^.

In a comparison of different tonometers following intrastromal corneal ring implantation, Elfwwal et al.^(27)^ reported that IOP measurements obtained with air puff tonometers were significantly lower than those measured with GAT and ORA-IOPcc. They considered that the results may be due to corneal rigidity after ICRS implantation. Conversely, in their investigation of the impact of ICRS implants (Keraring) on biomechanical parameters in eyes with keratoconus, Gorgun et al. found that corneal hysteresis and corneal resistance factor decreased during the early postoperative period (1st and 3rd months). However, these parameters returned to preoperative levels by the end of the 6th month, but further measurements between the 6th month and the 2nd year showed a gradual increase, although statistically insignificant^(28)^.

The limitations of our study were that IOP measurements by both devices may have been affected by corneal biomechanical properties (corneal hysteresis), which we could not evaluate in this study. Furthermore, we also did not evaluate the influence of refractive errors on the IOP measurements obtained with the three tonometers. Avitabile et al. evaluated the effect of refractive errors on IOP measurements using RbT and GAT and found RbT readings >2 mmHg in 17.9% (emmetropic), 13.3% (hypermetropic), 34.5% (myopic), and 7.6% (astigmatic) eyes^(29)^, indicating that the influence of refractive errors must be considered when interpreting IOP recordings with these tonometers.

Further studies should assess the correlation between the studied variables (number of segments, volume of implanted segments, variation in mean keratometry, and variation in asphericity) and the behavior of postoperative pressure indices.

In conclusion, our study results suggest that despite statistically significant differences among some postoperative periods, no differences were observed in clinical practice as the inherent variability of GAT must be considered.

Although no significant bias was observed between GAT and RbT and NCT, the 95% LOAs demonstrate a relatively wide range of differences between the methods, possibly preventing the use of GAT, RbT, and NCT from interchangeably, but using the same tonometer consistently during clinical follow-up is almost as important as the tonometer choice.

Further studies should assess the analysis of the correlation between the variation in IOP and corneal physical-structural parameters after ICRS implantation, which may provide more details about the behavior of postoperative pressure indices.
